# ﻿Redefining the megagenus *Erica* L. (Ericaceae): the contributions of E. G. H. Oliver and I. M. Oliver (née Nitzsche) to taxonomy and nomenclature

**DOI:** 10.3897/phytokeys.244.121705

**Published:** 2024-07-05

**Authors:** E. Charles Nelson, Michael D. Pirie, Dirk U. Bellstedt

**Affiliations:** 1 Tippitiwitchet Cottage, 255A Broadgate, Sutton St Edmund PE12 0LT, Spalding, UK; 2 University Museum, University of Bergen, Postboks 7800, NO-5020 Bergen, Norway; 3 Dept of Biochemistry, University of Stellenbosch, Stellenbosch, South Africa; † Deceased

**Keywords:** Ericoideae, nomenclature, taxonomy

## Abstract

The megagenus *Erica* L. (Ericaceae), as it is recognised today, includes 851 species of evergreen shrubs or small trees, the majority of which are endemic to the Cape Floristic Region of South Africa. From the first descriptions in Linnaeus’s *Genera plantarum*, a succession of authors ascribed the steadily accumulating numbers of known species to various of a total of 72 different genera. Until the latter half of the twentieth century, so called ‘minor genera’ such as *Philippia* Klotzsch and *Blaeria* L. were still recognised for many African species. The now uncontroversial inclusive circumscription of *Erica*, and a substantial proportion of its currently recognised species diversity, was conceptualised, described, and illustrated by the South African botanists E. G. H. (‘Ted’) Oliver and Inge M. Oliver in a succession of works published from 1964 to the present day. We review the historical development of generic delimitation in *Ericasens. lat.*, focusing on the contribution of the Olivers to the current state of systematic knowledge of the genus, and presenting an overview and complete lists of literature and of taxa that they authored.

## ﻿Introduction: historical conspectus

*Erica* L. (subfamily Ericoideae, family Ericaceae), as understood today, is one of the largest genera in the Angiospermae. World Flora Online (WFO; www.wfo.org) currently recognizes 851 included species (https://wfoplantlist.org/taxon/wfo-4000013772-2024-06; Oliver et al. 2024; Elliott et al. 2024). Within this concept of *Erica*, 72 genera that were designated and named at various times since 1753 have been subsumed – some of these were known informally as ‘minor genera’ following Phillips’s use of that phrase (Phillips 1944), until the major revision by E. G. H. Oliver ‘sank’ all of them into *Erica* (Oliver 2000a; Oliver and Oliver 2000a).

The taxonomic and nomenclatural history of *Erica* is complicated. Carl Linnaeus’s *Genera plantarum* (Linnaeus 1737: 110) and *Species plantarum* (Linnaeus 1753: 112, 352–356) are the starting points for modern classification and nomenclature (Oliver 2007; Jarvis 2007). In the first edition of *Genera plantarum*, Linnaeus (1737: 110 genus no. 312) defined *Erica* in just 51 Latin words, exclusively referring to the anatomy and morphology of the flowers, placing the genus in Class VIII ‘Octandria Monogynia’ based on the numbers of male (eight stamens) and female (one gynoecium) organs in each flower. (By way of contrast, the most recent definition of *Erica* (Oliver 2000a) exceeds 350 words (in English) (Suppl. material 1) (for a more succinct description, about 100 words, see Oliver 2004).)

In the first edition of *Species plantarum* Linnaeus described and named only 23 species of *Erica*. His artificial method of classification led him to nest the widespread, northern-hemisphere shrubby *Callunavulgaris* L. within *Erica* (as *Ericavulgaris* L.: Linnaeus 1753: 352 no. 1). On the same artificial basis, another *Erica*-like African shrub with only four stamens and a solitary gynoecium (‘Tetrandria Monogynia’), was placed by him in a separate genus named *Blaeria* L. (as *B.ericoides* L.: Linnaeus 1753: 112). Forty-eight of the binomial names published by Linnaeus in eight works produced during his lifetime – in *Species plantarum* (Linnaeus 1753) and its second edition (Linnaeus 1762), a dissertation defended by Jacob Printz entitled *Plantae rariores Africanae* (Linnaeus 1760), the tenth and twelfth editions of *Systema naturae* (Linnaeus 1759, 1767a), two editions of *Mantissaplantarum* (Linnaeus 1767b, 1771) and in a second dissertation *De Erica* (Linnaeus 1770) defended by Johan Adolph Dahlgren – are still in use in *Erica* (see entries in Jarvis 2007: 497–501). Since Linnaeus’s time, the number of taxa recognized as belonging to *Erica* has multiplied vastly, presenting today’s taxonomists with not a few difficulties in delimiting taxa at generic and subgeneric levels.

**Figure 1.**
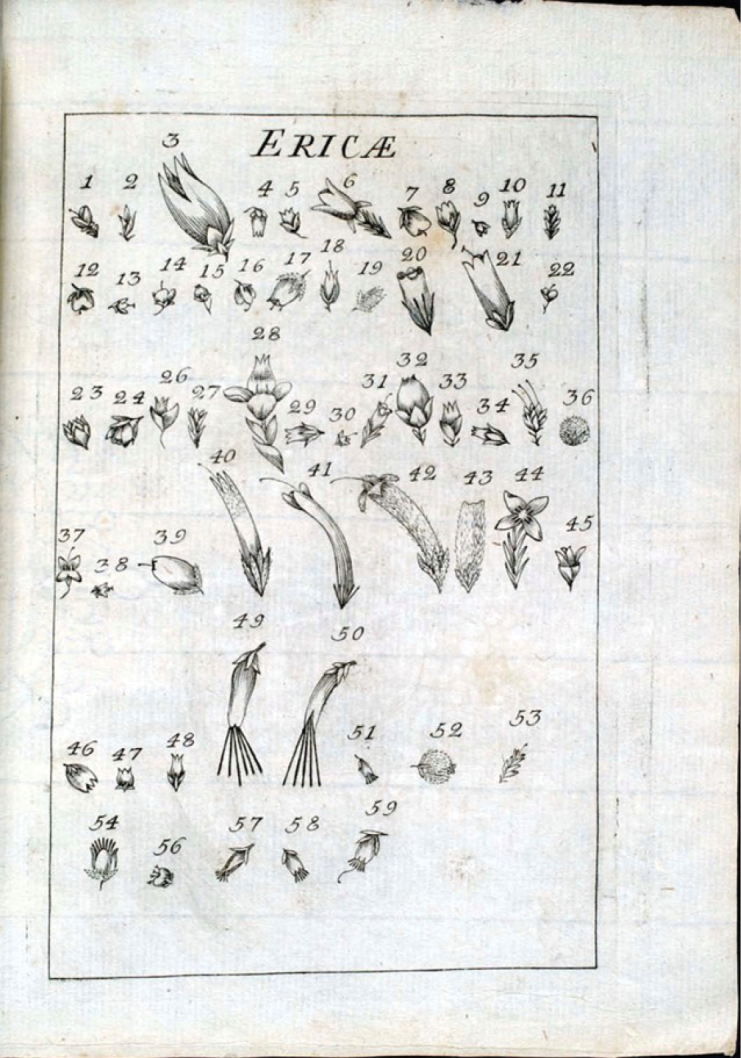
Flowers of *Erica* species known to Carl Linnaeus, from Linnaeus (1770).

Vegetatively, *Erica* species (commonly called heaths or heathers) are relatively similar, being shrubs or less frequently small trees with small, evergreen, linear-oblong (‘needle-like’) leaves arranged in whorls. The leaves often have revolute margins that can touch on the underside – this general type of leaf morphology is termed ‘ericoid’. As the individual taxa are so similar in foliage, taxonomists, since Linnaeus’s time, have traditionally relied on floral characteristics to demarcate subgeneric taxa and distinguish between species, rather than foliage morphology (for example, see Linnaeus 1770) (Fig. 1). Linnaeus (1753: 352, 354) used the presence/absence of a pair of awns at the base of each anther to subdivide the species he knew – ten species with awned anthers (‘Antheris bicornibus’) were separated from thirteen species with muticous (without awns) anthers (‘Anteris [*sic*] simplicibus obtusis emarginatis’). With additional species to accommodate, this scheme was modified to separate those species with included stamens – stamens that did not protrude beyond the mouth of the urn-shaped or tubular corolla – from others with exserted stamens (Linnaeus 1770) (Fig. 1). The number and morphology of the stamens in the flowers of shrubs that could be recognized as *Erica*-like continued to dominate *Erica* taxonomy until the end of the twentieth century. Additional characters linked with the morphology of the gynoecium, particularly the number of locules (ranging from one to eight) comprising the ovary and the number of ovules per locule, and whether the mature capsule was indehiscent or dehiscent, were employed in generic definitions.

The eighteenth-century and nineteenth-century botanists who worked to identify, name, and describe *Ericasens. lat.* species and who attempted to subdivide the numerous species into discrete genera were almost all based in Europe. A few had travelled and collected in Africa. Thus, most of their work entailed examining a relatively small number of pressed and dried specimens. Access to living material was largely limited to the species indigenous in the northern hemisphere, and to the relatively small number of cultivated plants maintained in European gardens. While there was a ‘mania’ for cultivating southern African *Erica* (the so-called ‘Cape heaths’) particularly during the first part of the nineteenth century (Nelson and Oliver 2004; Nelson and Pirie 2022), the plants grown were neither representative of the genus throughout its geographical range nor of its complex morphology. Small-flowered wind-pollinated heathers, for example, were not fashionable and did not attract gardeners. Besides, artificial and accidental cross-pollination in cultivation had produced a plethora of hybrids that tended to be preferred by horticulturists. The major attempts to produce monographs about *Erica* were thus both incomplete and compromised.

The only universal treatment available before 1900 was George Bentham’s contribution to the seventh volume of "Agustin Pyramus de Candolle’s Prodromus systematis naturalis regni vegetabilis" published in 1839 (Bentham 1839; see also Nelson 2005). In late 1906, when the Ericaceae section (parts 1–3) of the fourth volume of "Flora Capensis" (Bolus et al. 1905 [–1906]), covering the plants of southern Africa, was completed, an integrated treatment of diverse *Erica* flora of the Cape Floristic Region became available. This treatment of *Erica* (in a restricted sense) had been produced by Francis Guthrie (1831–1899) and Harry Bolus (1834–1911) (with assistance latterly from his future daughter-in-law, Harriet Margaret Louisa Kensit (*olim* Bolus) (1877–1970)), botanists who lived in the Cape Province of South Africa where *Erica* species are indigenous and numerous. Regarding their concept of *Erica*, Guthrie and Bolus (1905: 5) commented that ‘The genus is remarkable for an unusual degree of variability in the form of almost all its organs. It is therefore one difficult of definition as to its species and of arrangement into satisfactory natural groups.’ They had subdivided the 469 southern African species of *Erica* that they recognized into five subgenera and 41 sections, as well as accepting that close to 160 other species should be placed outside *Erica* in 22 ‘minor genera’ (Phillips 1944: 69; Oliver 2000b: 55). These ‘minor genera’, six being monotypic, were treated by Nicholas Edward Brown (1849–1934), a botanist based at the Royal Botanic Gardens, Kew (Brown 1906). However, many other species native elsewhere in tropical and subtropical Africa and on Atlantic and Indian Ocean islands were not integral in this treatment. Edwin Percy Phillips (1884–1967) was the next to tackle the complexities of the African *Ericasens. lat.* His treatments of Ericaceae (Phillips 1926, 1944, 1951) also include *Vaccinium* L. (Vaccinioideae), represented by a single species now treated as *V.exul* Bolus, which occurs on the Eastern Escarpment (Mpumalanga and Limpopo Provinces), in Swaziland and in Malawi, in habitats above 1,200m altitude (Bester 2015). All other Ericaceae in South Africa represent *Ericasens. lat.* In his paper ‘Notes on the minor genera of Ericaceae’, Phillips (1944) reduced the 22 ‘minor genera’ retained in "Flora Capensis" to six, a scheme he maintained in the second edition of his "The genera of South African flowering plants" (Phillips 1951). Emphasising that he considered that the number of ovules per locule was ‘a more important character than the number of ovary-chambers [locules]’ in any attempt at a natural classification of the South African Ericoideae, Phillips (1944, 1951) retained only *Erica* L., *Blaeria* L., *Eremia* D.Don, *Sympezia* Licht., *Scyphogyne* Brongn., *Salaxis* Salisb. and *Lagenocarpus* Klotzsch.

## ﻿Taxonomic studies of E. G. H. Oliver and I. M. Oliver

**Figure 2.**
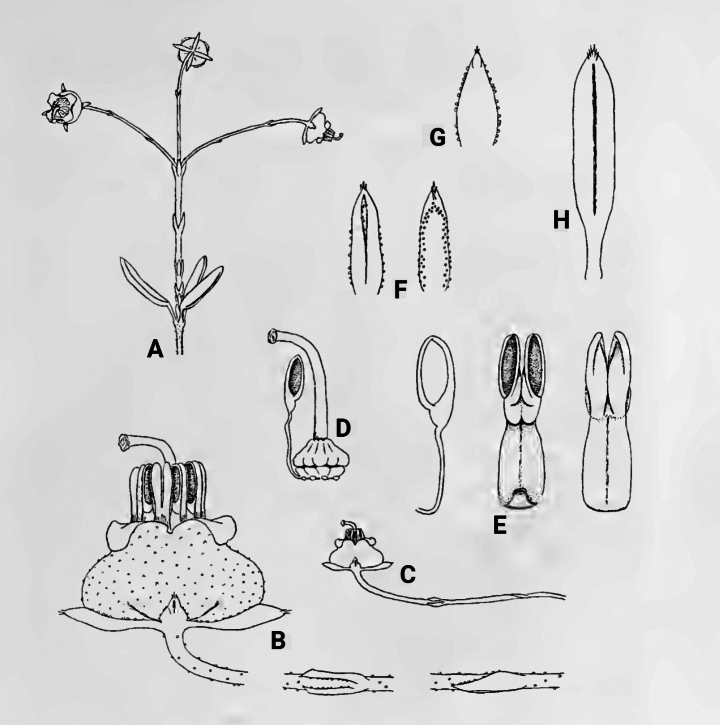
*Ericaoliveri* H.A. Baker, drawn by E. G. H. Oliver (Baker 1962): “The species has been named after Mr. E. G. H. Oliver who is making a study of the genus and of the minor genera and has already collected a number of hitherto unknown species and seems destined to add many more to the 600 or so [*sic*] already described.” **A** spring **B, C** flower **D** gynoecium **E** anther **F** sepals **G** bract **H** leaf.

Edward (‘Ted’) George Hudson Oliver became fascinated by the diversity of Cape flora and especially fynbos vegetation after he enrolled as an undergraduate, to study zoology, at the University of Cape Town in the late 1950s. His attention was soon diverted from animals, and he became ‘obsessed’ by the Cape heaths because ‘they were small and delicate with a seemingly infinite variety of shapes and colours’ (Oliver 2000b). He was already collecting *Erica* in the field and making discoveries and, according to Colonel Hugh Arthur Baker (1896–1976) when he named *E.oliveri* (Fig. 2) in July 1962, ‘Mr. E. G. H. Oliver … seems destined to add many more to the 600 or so [*Erica* species] already described’ (Baker 1962: 198).

Following graduation, he undertook a study of *Acrostemon*, one of the ‘minor’ genera, presenting his Master’s thesis in October 1964. In this (Oliver 1964) he noted ‘complete overlap between *Acrostemon* Klotzsch and *Hexastemon* Klotzsch allowing no character for the separation of the two genera’ and therefore proposed to incorporate *Hexastemon* in *Acrostemon*. Likewise, comparing the monotypic genus *Arachnocalyx* Klotzsch, which had been designated by Robert Harold Compton (1886–1979) (Compton 1935), with the single species *Acrostemonviscidus* N.E.Br., Oliver found ‘much closer similarity’ between *Arachnocalyxeriocephalus* (Klotzsch) N.E.Br. and *Acrostemonviscidus* ‘than had previously been suspected’ (Oliver 1964: 105). To gain wider insights into the patterns of morphological similarities and differences, the species then assigned to *Syndesmanthus* Klotzsch and *Simochilus* Klotzsch were investigated too, and again Oliver (1964: 105) noted that ‘a certain degree of intergrading occurs in the differentiating characters between the three genera to an extant where it becomes difficult to distinguish and place species into any one of the genera.’ Discussing the six ‘minor genera’ that Phillips had retained, Oliver (1964: 115) concluded that Phillips’s scheme was ‘completely artificial’ and did not ‘reflect the correct relationships between the genera which [had] been reduced to synonymy. … With the addition of new and well collected material, a reconsideration of the generic boundaries will certainly be necessary in a future revision … [T]he problem of generic distinction will have to be worked out carefully and thoroughly.’ At that time, some of the ‘minor genera’ were still regarded as monospecific, including the European endemic *Bruckenthalia* Rchb. and the last African ‘minor’ genus to be described, *Stokoeanthus* E.G.H. Oliv. (Oliver 1976a). Commenting on his decision to describe and name that new monotypic genus, he stated (Oliver 1976a):

The relationship of [*Stokoeanthus*] appears to me to be with *Erica* and *Blaeria* and to some extent with *Eremia*, but it does not fit into any of them as presently constituted. From *Erica* it differs in having 4 stamens and a 2-celled ovary, from *Blaeria* in having 2 cells to the ovary and from *Eremia* in having 4 stamens and more than 1 ovule per cell. To change the generic limits of any of these genera to force the inclusion of the new taxon would, in my opinion, be impracticable and would cause repercussions in the relationships of and differences between many other genera of the Ericoideae.

Thus, Ted laid down the basis for the work that consumed his time for the next 35 years, culminating in the elimination of all the ‘minor genera’ (Oliver 1987; 1988; 1992; 1993b; 1993a; 1993c; 1994; 1996; 2000a) and the subsuming of all their species into the megagenus *Erica* (Schumann et al. 1992: 244; Oliver 2000a; Oliver and Oliver 2000a). By examining many more plants than had been available to preceding botanists, he noticed clearly overlapping characters in the ‘minor genera’ and apparently discontinuous variation coalescing through various intermediate states, a good example being the capacity of the mature capsule to dehisce.

**Figure 3.**
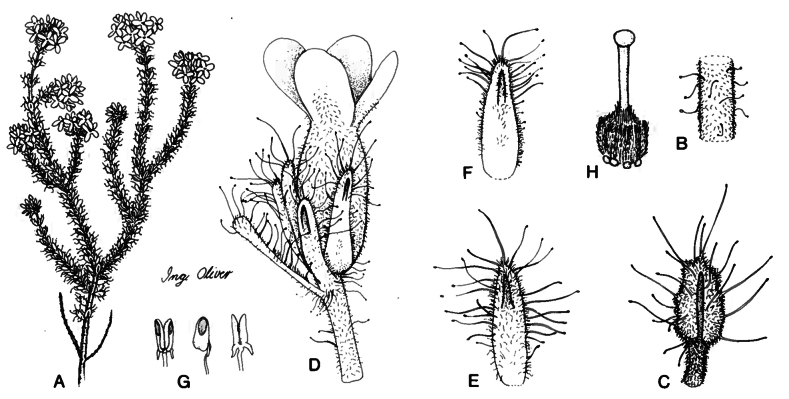
*Ericaingeana* E.G.H. Oliver, drawn by Inge M. Oliver (Oliver and Oliver 1991) **A** flowering branch **B** branch **C** leaf **D** flower **E** bract **F** sepal **G** anther, front, side and back views **H** gynoecium.

From about 1974, Oliver was assisted in his work on *Erica*, especially the ‘minor genera’, by Inge Magdalene Nitzsche (1947–2003), who had studied botany and zoology at the University of Cape Town (1967–1969) and also had a diploma in fine arts (1971–1972). They married in February 1974. Inge’s remarkable pen-and-ink drawings of anatomical and morphological details of the species (often not signed) were to be an integral component of papers about *Erica* (and the ‘minor genera’) published from 1976 onwards: early (unsigned) examples of Inge Oliver’s extraordinary illustrations were published in the paper ‘revising’ *Eremia* and *Eremiella* (Oliver 1976b: fig. 2, p. 34 *Eremiatotta* (Thunb.) D.Don; fig. 9, p. 40 *E.curvistyla* (N.E.Br.) E.G.H. Oliv.; fig. 14, p. 44 *E.brevifolia* Benth.) (for the eponymous *Ericaingeana* E.G.H. Oliver, see Oliver and Oliver 1991: 140–142 (Fig. 3)).

**Figure 4.**
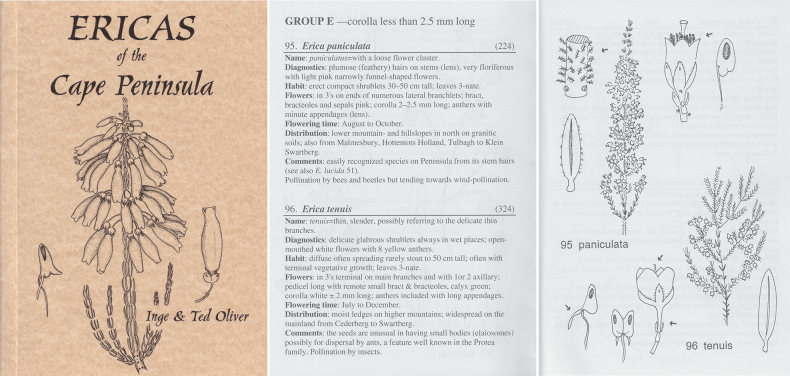
*Ericas of the Cape Peninsula* by Inge and Ted Oliver (2000, published by the Protea Atlas Project, National Botanical Institute, Cape Town); left (cover: 106 × 148mm): *E.mammosa*, pen-and-ink drawing by Inge Oliver: text page and accompanying illustrations by Inge Oliver, showing *E.paniculata* and “*E.tenuis*” (now *E.oliveranthus* E.C.Nelson & Pirie; Nelson et al. 2023). Reproduced with permission.

In 1988, a grant enabled Inge to be employed as research assistant in the BRI Herbarium at the University of Stellenbosch ‘to help with all the technical work – the numerous dissections, drawings and recording of all the details of variations in the plants. This … helped tremendously with the research and … Inge’s work also helped with the finalisation of the analvses of the minor ericaceous genera …’ (Oliver 2000b; Nelson 2004). Ted Oliver submitted his doctoral thesis to the University of Cape Town in 1999 (Oliver 1999) and published the monograph on the ‘minor genera’, integrating all of them into the redefined megagenus *Erica* in 2000. "Field guide to the Ericas of the Cape Peninsula" (Oliver and Oliver 2000b), a handy, pocketable manual, represents another aspect of these collaborative studies, making available an identification aid, illustrated with simple line drawings (Fig. 4), for naturalists in general.

Having come into contact with Colonel H. A. Baker by the early 1960s, Ted Oliver became co-author with Baker of "Ericas of South Africa" (Baker and Oliver 1967), illustrated with botanical paintings by the South African botanical artist Irma von Below (1920–1984) and others. Two decades later he started to collaborate with ‘Dolf’ (Adolf Wilhelm Stander) Schumann (1918–2001) and Gerhard P. K. Kirsten (1932–2000) on a profusely illustrated photographic monograph "Ericas of South Africa" (Schumann et al. 1992) in which the amalgamation into the megagenus *Erica* of the last of the minor genera was announced, because they have ‘so much in common … that their species should also be regarded as ericas [*sic*]’ (Schumann et al. 1992: 244)

Writing for The Heather Society (of which he was an honorary member) in 2000, Ted noted that:

"Having begun work on the Ericaceae some 40 years ago as a student, I now find that I am getting to know the group properly, the more so recently because of the rapidly increased knowledge that Inge has also acquired. I am no longer a single person struggling in a “sea of ericas”. There are now two of us to discuss the problems of species de-limitation, species evolution and the phylogeny, ecology or phytogeography of this fascinating genus. There are quite a number of new species to be described and surprisingly, other un-described species are still being brought in. The biggest problem ahead is that of sub-generic classification. The new techniques of DNA analysis will help to throw some light on this problem, which is now being tackled by a group of international experts with material that I am supplying for them."

In Table 1, we present an abridged timeline of the careers of Ted and Inge with selected publications, eponyms, and other noteworthy milestones.

**Table 1.** Milestones in the careers of E. G. H. Oliver and I. M. Oliver, relating to their studies, separately and jointly, of the megagenus *Erica* (1937–2024).

	E. G. H. (“Ted”) Oliver	Inge Magdalene Oliver (née Nitzsche)
	1938–	1947–2003
1959	undergraduate, University of Cape Town (–1962)	
1962	commences Masters degree, in Bolus Herbarium; H. A. Baker describes and names *Ericaoliveri*	
1964	submitted Masters thesis "Taxonomic studies in the genus *Acrostemon* Kl. and related genera"; M.Sc. awarded	
	Curator, Government Herbarium, University of Stellenbosch	
1967	"*Erica* of South Africa" published; co-author Colonel Hugh A. Baker	undergraduate, University of Cape Town (–1969)
1967	South African Botanical Liaison Officer, Royal Botanic Gardens, Kew, UK	
1970	returned to herbarium, University of Stellenbosch	
1971		commenced Fine Arts diploma
1972: December	elected honorary member of The Heather Society	
1974: February	marriage	
1975	Head of Herbarium Services and Curator National Herbarium, Pretoria	
1976		first illustrations (unsigned) of Erica published in E. G. H. Oliver, ‘Studies in the Ericoideae. I. The genera *Eremia* and *Eremiella*’, Bothalia 12 (1).
1981	returns to Government Herbarium, University of Stellenbosch	
1988		research assistant at herbarium, University of Stellenbosch
1991	*Ericaingeana* named and described in earliest co-authored research paper (‘Studies in the Ericoideae (Ericaceae). VIII. New species in *Erica*, section Pseuderemia, from southern Africa’. Bothalia 21 (2))	
1992	"Ericas of South Africa": published; co-authors Dolf Schumann and Gerhard Kirsten	
1999	submits doctoral thesis ‘Systematic studies in the Tribe Ericeae (Ericaceae–Ericoideae)’	
2000	Ph.D. awarded; monograph (Systematics of Ericeae (Ericaceae: Ericoideae) species with indehiscent and partially dehiscent fruits. "Contributions from the Bolus Herbarium" no. 19) published; contained 84 full-page illustrations by Inge M. Oliver	"Field guide to the Ericas of the Cape Peninsula" published; 104 species illustrated
2003: July		deceased
2010	visited Madagascar (with group including DUB & MDP; Heathers 8: 47–54. 2011)	
2012	Genus *Erica* An identification aid version 3.00 published (Contributions from the Bolus Herbarium 22); co-author Nigel Forshaw [version 4.00 published 2024]	
2014	visited Mauritius (Heathers 11: 38–42. 2014)	

Although big plant genera have expanded and contracted over time (Frodin 2004), it is relatively unusual for twentieth-century taxonomists working on morphology alone to change generic delimitations in favour of fewer, larger genera. More often, the emphasis has been placed on morphological differences in particular groups, without necessarily addressing the coherence of groups from which they are split (Humphreys and Linder 2009). Oliver’s ‘megagenus’ concept for *Erica* (Oliver 2012; Oliver and Forshaw 2012) reflected a global understanding of the group that pre-empted subsequent molecular research in which he was instrumental. Phylogenetic trees including more than 40% (Pirie et al. 2011) and 60% (Pirie et al. 2016.) of the species diversity clearly showed that the ‘minor genera’ are nested within – and indeed scattered across – the redefined mega-genus *Erica*.

One example, *Philippia* Klotzsch, was characterised largely by reduced flowers without brightly coloured corollas or nectaries, but with greatly expanded stigmas (as illustrated in Oliver 1988: 4 & 5), together interpreted as a wind-pollination syndrome (Rebelo et al. 1985). Transition to wind pollination was shown to have occurred several times in *Erica* as then defined (Pirie et al. 2011), with similar characteristics shared by the wind-pollinated ‘minor genera’ such as *Salaxis* Salisb., *Coccosperma* Klotzsch and *Ericinella* Klotzsch (Oliver 1994, 2000a) and many individual species scattered within *Ericasens. str.*

**Figure 5.**
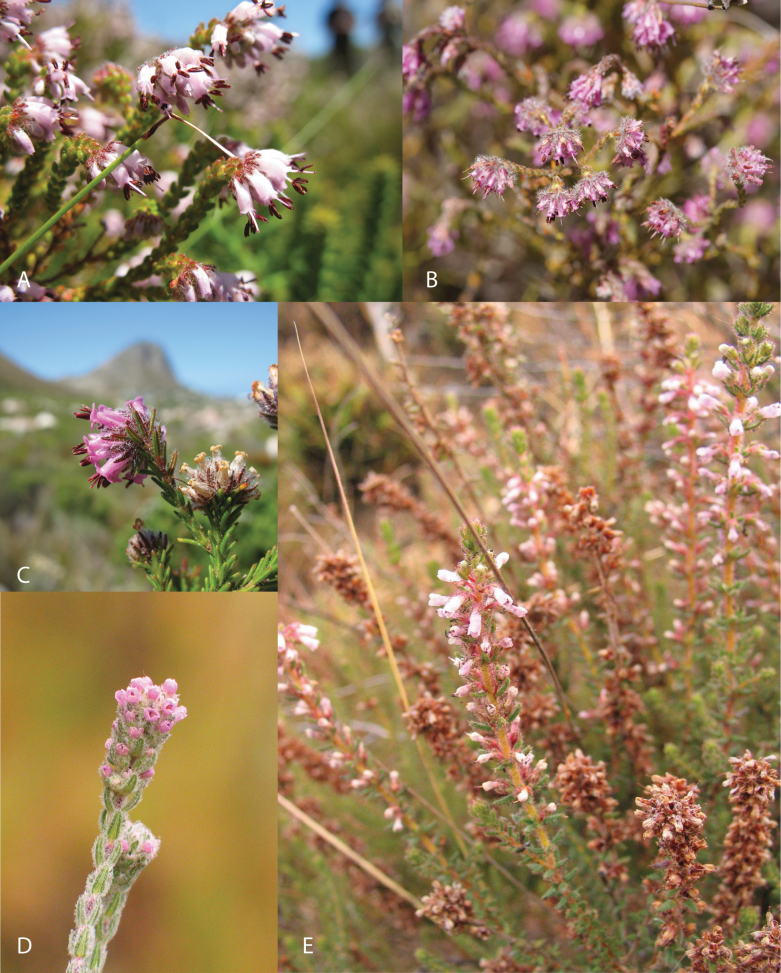
Examples of the former genus *Blaeria*. Cape species included in *Erica* by Ted Oliver: **A***E.ericoides***B***E.russakiana*; and **C***E.barbigeroides*, and tropical East African species included in *Erica* by Beentje (2006): **D***E.filago* and **E***E.silvatica*. Photos **A** MDP **B–E** Berit Gehrke.

*Blaeria* L., had been defined as including those species with four, rather than eight, stamens (Linnaeus 1753, Bentham 1839; Brown 1906; Phillips 1926, 1944; Oliver 1975, 1993b) but this definition was not subsequently applied consistently. Phillips (1944, 1951) included species with four, six or eight stamens in the ‘minor genera’ *Coccosperma* (4–8 stamens) and *Philippia* (6–8 stamens), but the numbers were also not consistent: four stamens can arise within an individual species usually characterised by having eight (for example, *Ericafiliformis* Salisb., *E.blaerioides* E.G.H. Oliv., *E.arborea* L., *E.woodii* Bolus, *E.pleiotricha* S. Moore; Oliver 1993b). As anticipated by Oliver (1993b), neither the ‘minor genus’ *Blaeria*, nor species of *Ericasens. lat.* possessing only four stamens, proved to represent monophyletic groups, and former *Blaeria* species, such as *E.ericoides* (L.) E.G.H. Oliv., *E.barbigeroides* E.G.H. Oliv. and *E.russakiana* (Klotsch ex Walp.) E.G.H. Oliv. from the Cape and the tropical East African species *E.filago* (Alm & T.C.E. Fr.) Beentje and *E.silvatica* (Welw. ex Engl.) Beentje (included in *Erica* by Beentje (2006), explicitly following Oliver’s precedent; Fig. 5), proved to be distantly related.

With the megagenus concept already firmly embedded, the Olivers set out to revise systematically groups of species following the arrangement in "Flora Capensis" (Guthrie and Bolus 1905; Brown 1906). Two such works were accomplished (Oliver and Oliver 2002, 2005), and many more might have been expected but for the early death of Inge in 2003 (Nelson 2004). Ted continued work on the *Erica* Identification Aid, which includes many of Inge’s informal sketches (Oliver and Forshaw 2012; Oliver et al. 2024) and provides a route towards species identifications that is also accessible to non-specialists. His support of molecular research from 2008 onwards has included analyses of European species (Mugrabi de Kuppler et al. 2015) and improvements to species delimitation in Cape groups (Pirie et al. 2017) as well as broad scale phylogenetic and biogeographic analyses (Pirie et al. 2016; 2019; 2024) reflecting his broader interests in patterns and origins of Cape plant diversity (Oliver et al. 1983).

## ﻿Conclusion

**Figure 6.**
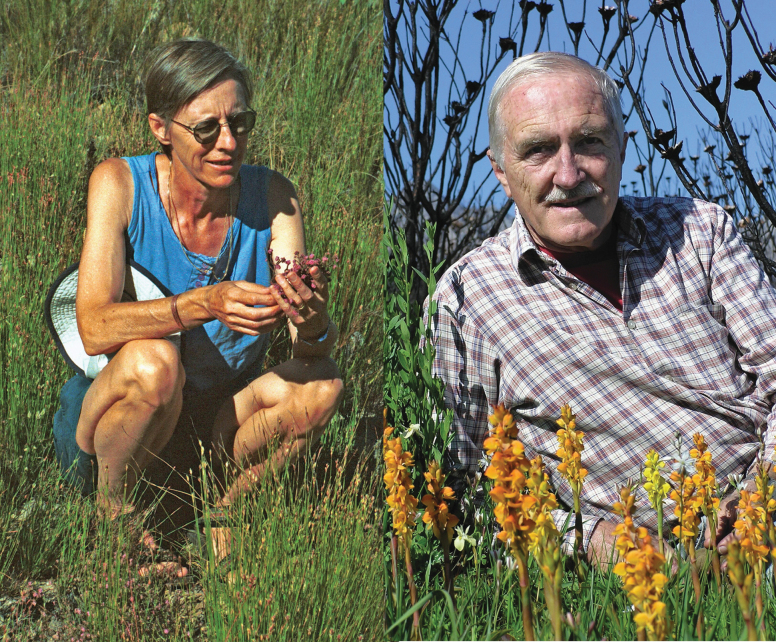
Inge M. Oliver (left) and E. G. H. (Ted) Oliver (photos provided by Tessa Oliver).

By the end of the twentieth century, following almost 250 years of botanical exploration especially in the Cape Floristic Region of South Africa, the number of species of heaths and heathers known to botanists had exceeded eight hundred (Oliver and Oliver 2000a) – many hundreds more binomial names had been published (Nelson and Small 2004 [–2005]). By early 2024 the total number of accepted *Erica* species was 851 (Elliott et al. 2024). Not only is *Erica* confirmed as one of the largest genera of Angiospermae, it is one of the most widely distributed, its species ranging across more than 100 degrees of latitude from northern Norway to the Cape Floristic Province in South Africa, and, straddling the Equator, for almost 90 degrees of longitude from the Azores (31°W) in the Atlantic Ocean eastwards to islands in the Indian Ocean including Madagascar, the Mascarenes and Mauritius (57°E) (Oliver 1994, 2011, 2014). At this time, of the currently accepted species, 94 were described and named as new to science by Ted Oliver, and many of these were first collected by him too, while a further 206 are combinations and replacement binomials published under his name, as sole or joint author (Suppl. material 2). This includes not only Cape diversity, but also taxa from Tropical East Africa and Madagascar (Dorr and Oliver 1999a, 1999b), and the Mascarenes (Oliver 1993a). Ted and Inge Oliver (pictured in Fig. 6) jointly described and named 16 novel species of *Erica* (Table 2). Inga’s illustrations occurred in many of the papers published since 1974 and (as noted) in the *Erica* Identification Aid (Oliver et al. 2024). A full list of their papers on *Erica* is presented in Suppl. material 3.

**Table 2.** New species named by E. G. H. Oliver & I. M. Oliver.

*Ericaamalophylla*	E.G.H.Oliv. & I.M.Oliv.	wfo-4000013772
*Ericaannalis*	E.G.H.Oliv. & I.M.Oliv.	wfo-4000013772
*Ericacavartica*	E.G.H.Oliv. & I.M.Oliv.	wfo-4000013772
*Ericaceraria*	E.G.H.Oliv. & I.M.Oliv.	wfo-4000013772
*Ericacroceovirens*	E.G.H.Oliv. & I.M.Oliv.	wfo-4000013772
*Ericagerhardii*	E.G.H.Oliv. & I.M.Oliv.	wfo-4000013772
*Ericahebdomadalis*	E.G.H.Oliv. & I.M.Oliv.	wfo-4000013772
*Ericajananthus*	E.G.H.Oliv. & I.M.Oliv.	wfo-4000013772
*Ericajugicola*	E.G.H.Oliv. & I.M.Oliv.	wfo-4000013772
*Ericalithophila*	E.G.H.Oliv. & I.M.Oliv.	wfo-4000013772
*Ericapetrusiana*	E.G.H.Oliv. & I.M.Oliv.	wfo-4000013772
*Ericaprolata*	E.G.H.Oliv. & I.M.Oliv.	wfo-4000013772
*Ericapsittacina*	E.G.H.Oliv. & I.M.Oliv.	wfo-4000013772
*Ericaschelpeorum*	E.G.H.Oliv. & I.M.Oliv.	wfo-4000013772
*Ericaumbratica*	E.G.H.Oliv. & I.M.Oliv.	wfo-4000013772
*Ericaviridimontana*	E.G.H.Oliv. & I.M.Oliv.	wfo-4000013772

No single author has had greater impact on the taxonomy of *Erica*/Ericeae than Ted Oliver in numbers of new species (as predicted by his mentor, H. A. Baker, in 1962), but this contribution must be interpreted as part of a team effort of two enormously talented botanists. Their combined contribution both of improved knowledge of alpha taxonomy and of broad understanding of the structure of that diversity is fundamental to, and will have a lasting influence on, all future developments in the field.

## ﻿Additional information

### Author ORCIDs

E. Charles Nelson https://orcid.org/0000-0002-3909-4388

Michael D. Pirie https://orcid.org/0000-0003-0403-4470

Dirk U. Bellstedt https://orcid.org/0000-0002-6376-4855

### Data availability

All of the data that support the findings of this study are available in the main text or Supplementary Information.

## Supplementary materials

Supplementary material 1 Generic description of *Erica* by E. G. H. Oliver (2000a; from Nelson 2011: 100) Data type docx https://binary.pensoft.net/file/1085785This dataset is made available under the Open Database License (http://opendatacommons.org/licenses/odbl/1.0/). The Open Database License (ODbL) is a license agreement intended to allow users to freely share, modify, and use this Dataset while maintaining this same freedom for others, provided that the original source and author(s) are credited.E. Charles Nelson, Michael D. Pirie, D. U. Bellstedt

Supplementary material 2 A complete list of *Erica**s.l.* taxa at all ranks authored by E.G.H. Oliver and I.M. Oliver Data type xlsx Explanation note Including taxa new to science, replacement names, and new combinations, with synonymy and bibliographic data, derived from the World Flora Online (Elliott et al. 2024). https://binary.pensoft.net/file/1085786This dataset is made available under the Open Database License (http://opendatacommons.org/licenses/odbl/1.0/). The Open Database License (ODbL) is a license agreement intended to allow users to freely share, modify, and use this Dataset while maintaining this same freedom for others, provided that the original source and author(s) are credited.E. Charles Nelson, Michael D. Pirie, D. U. Bellstedt

Supplementary material 3 A complete list of *Erica* related publications authored by E.G.H. Oliver and I.M. Oliver Data type docx https://binary.pensoft.net/file/1085787This dataset is made available under the Open Database License (http://opendatacommons.org/licenses/odbl/1.0/). The Open Database License (ODbL) is a license agreement intended to allow users to freely share, modify, and use this Dataset while maintaining this same freedom for others, provided that the original source and author(s) are credited.E. Charles Nelson, Michael D. Pirie, D. U. Bellstedt
