# Supplementary material for: ﻿Redefining the megagenus Erica L. (Ericaceae): the contributions of E. G. H. Oliver and I. M. Oliver (née Nitzsche) to taxonomy and nomenclature
Source: PhytoKeys. 2024 Jul 5;244:39–55. doi: 10.3897/phytokeys.244.121705 (PMC11245645; doi:10.3897/phytokeys.244.121705)
Supplement: Supplementary material 1 — Generic description of Erica by E. G. H. Oliver (2000a; from Nelson 2011: 100) [file phytokeys-244-039_article-121705__-s001.docx]

**Appendix 1:** Generic description of *Erica* by E. G. H. Oliver (2000a; from Nelson 2011: 100)

**ERICA L.**, Species plantarum **1**: 352 (1753).

(For more comprehensive synonymy see Oliver 2000a: 98–100).

Prostrate shrublets, erect shrubs, or trees to 15–20m tall. Leaves 3- or 4-nate in whorls, occasionally 6-nate, rarely opposite or spirally arranged, mostly linear-oblong to ovate, trigonous, with revolute margins almost touching on underside (ericoid), less commonly broad, flat and open-backed but then less than 10mm × 5 mm, sometimes very reduced and scale-like or enlarged coloured and sepaloid below the inflorescences. Flowers always axillary, in 1–12 whorls variously arranged in elongate racemose to umbellate florescences, either at ends of most leafy lateral branches or very reduced lateral branchlets or only the main branches, sometimes forming dense synflorescences; pedicel usually present, sometimes very reduced, very rarely non-existent; bract always present, on the main axis or partially recaulescent to fully recaulescent forming the abaxial lobe/segment of calyx; bracteoles 1 or 2, or absent, sometimes included in the calyx as the lateral lobes/segments. Calyx hypogynous, (1–)4–5-partite or -lobed, small and leaf-like or large and showy, rarely longer than corolla, sometimes enlarging and thickening considerably in fruiting stage. Corolla persistent, hypogynous, (2–)4–5-lobed, tubular, ampullaceous, urceolate, globose, ovoid, campanulate, cyathiform, obconic or funnel-shaped, small to large and brightly coloured, dull coloured in most wind-pollinated species. Stamens (3–)8–10, free or completely fused, included or exserted; anthers dorsifixed or basifixed, with or without simple or elaborate dorsal appendages, thecae partly united or sometimes free, with small subterminal pores or pores sometimes as long as thecae; pollen shed as tetrads or sometimes as monads. Ovary (1–)4(–8)-locular, with 1–180 ovules per locule, sessile or stalked, nectaries usually prominent around base, sometimes absent in wind-pollinated species; style filiform to cylindrical, rarely non-existent, exserted or included; stigma/style complex simple-truncate, capitate, peltate, cyathiform or funnel-shaped and considerably enlarged in wind-pollinated species, rarely distinctly 4-lobed. Fruit mostly a dehiscent loculicidal capsule, sometimes a dry berry or drupe, pericarp hard and woody to very thin and papery; seeds with thick testa, mostly alveolate, some smooth or papillate, in indehiscent fruits the testa thick to very thin, transparent or almost non-existent; pits in inner periclinal and anticlinal walls present or absent. 2n = 24, 36.
