# Supplementary material for: ﻿Redefining the megagenus Erica L. (Ericaceae): the contributions of E. G. H. Oliver and I. M. Oliver (née Nitzsche) to taxonomy and nomenclature
Source: PhytoKeys. 2024 Jul 5;244:39–55. doi: 10.3897/phytokeys.244.121705 (PMC11245645; doi:10.3897/phytokeys.244.121705)
Supplement: Supplementary material 3 — A complete list of Erica related publications authored by E.G.H. Oliver and I.M. Oliver [file phytokeys-244-039_article-121705__-s003.docx]

**Appendix 3:** A complete list of publications relating to the genus *Erica* (Ericaceae) authored by E.G.H. Oliver and I.M. Oliver.

Oliver EGH (1964) Taxonomic studies in the genus *Acrostemon* Kl. and related genera. Master of Science thesis. Faculty of Science, University of Cape Town Available from: <http://hdl.handle.net/11427/39023> (November 10, 2023).

Baker HA, Oliver EGH (1967) Ericas in Southern Africa. With Paintings. Purnell, Cape Town.

Oliver EGH (1967) Three new species of *Erica*. Journal of South African Botany 33: 197–204. Available from: <https://archive.org/details/journalofsouthaf33unse/page/196/mode/2up>.

Oliver EGH (1969) Cape ericas. Journal of the Royal Horticultural Society 94: 341.

Oliver EGH (1972a) *Erica*, a remarkable genus. Journal of the Botanical Society of South Africa 58: 57–60.

Oliver EGH (1972b) *Erica oatesii*. Flowering Plants of Africa 42: 1644.

Oliver EGH (1972c) *Erica vallis-aranearum*. Flowering Plants of Africa 42: 1680.

Oliver EGH (1973) Ericaceae. *Erica krugeri*. Bothalia 11: 115–116. <https://doi.org/10.4102/abc.v11i1/2.1984>

Oliver EGH (1975) Ericaceae. In: Dyer RA (Ed.), The genera of South African flowering plants 1. Botanical Research Institute, Pretoria, 429–439.

Oliver EGH (1976a) Studies in the Ericoideae. I. The genera *Eremia* and *Eremiella*. Bothalia 12: 29–48. <https://doi.org/10.4102/abc.v12i1.1369>

Oliver EGH (1976b) Ericaceae. A new species of *Erica* from the Bredasdorp district. Bothalia 12: 57–59. <https://doi.org/10.4102/abc.v12i1.1373>

Oliver EGH (1976c) Studies in the Ericoideae. II. The new genus *Stokoeanthus*. Bothalia 12: 49–52. <https://doi.org/10.4102/abc.v12i1.1370>

Oliver EGH (1977a) Analysis of the Cape Flora. Proceedings of the National Weeds Conference of South Africa.

Oliver EGH (1977b) The identity of *Erica flavisepala*. Bothalia 12: 195–197. <https://doi.org/10.4102/abc.v12i2.1395>

Oliver EGH (1980a) *Erica*, eine bemerkenswerte Gattung des Kaplandes. In: Blumenparadiese und botanische Gärten der Erde. Pinguin-Verlag, Innsbruck, 135–138.

Oliver EGH (1980b) Studies in the Ericoideae. III. The genus *Grisebachia*. Bothalia 13: 65–93. <https://doi.org/10.4102/abc.v13i1/2.1292>

Oliver EGH (1981) Two new species of Ericoideae. Bothalia 13: 446–449. <https://doi.org/10.4102/abc.v13i3/4.1341>

Oliver EGH, Linder HP, Rourke JP (1983) Geographical distribution of present-day Cape taxa and their phytogeographical significance. Bothalia 14: 427–440. <https://doi.org/10.4102/abc.v14i3/4.1189>

Oliver EGH (1984a) Studies in the Ericoideae. IV. New species and some taxonomic and nomenclatural changes in the Cape Flora Region. South African Journal of Botany 3: 267–284. Available from: <https://core.ac.uk/reader/82239850> (November 10, 2023).

Oliver EGH (1984b) A new species of *Philippia*, from the Drakensberg. Bothalia 15: 550–551. <https://doi.org/10.4102/abc.v15i3/4.1842>

Rebelo AG, Siegfried WR, Oliver EGH (1985) Pollination syndromes of *Erica* species in the south-western Cape. South African Journal of Botany 51: 270–280. <https://doi.org/10.1016/S0254-6299(16)31657-X>

Oliver EGH (1986a) Ericaceae. A new species of *Ericinella* from the southern Drakensberg. Bothalia 16: 46–48. <https://doi.org/10.4102/abc.v16i1.1064>

Oliver EGH (1986b) The identity of *Erica vinacea* and notes on hybridization in *Erica*. Bothalia 16: 35–38. <https://doi.org/10.4102/abc.v16i1.1058>

McDonald DJ, Oliver EGH (1987) *Erica barrydalensis* L.Bol. - re-discovery of a rare species. Veld & Flora 73: 101–102. <https://doi.org/10.10520/AJA00423203_3833>

Oliver EGH (1987a) Studies in the Ericoideae (Ericaceae). V. The genus *Coilostigma*. Bothalia 17: 163–170. <https://doi.org/10.4102/abc.v17i2.1025>

Oliver EGH (1987b) Studies in the Ericoideae (Ericaceae). VII. The placing of the genus *Philippia* into synonymy under *Erica*; the southern African species. South African Journal of Botany 53: 455–458. <https://doi.org/10.1016/S0254-6299(16)31379-5>

Oliver EGH (1988) Studies in the Ericoideae (Ericaceae). VI. The generic relationship between *Erica* and *Philippia* in southern Africa,. Bothalia 18: 1–10. <https://doi.org/10.4102/abc.v18i1.975>

Oliver EGH (1989) The Ericoideae and the southern African heathers. Botanical Journal of the Linnean Society 101: 319–327. <https://doi.org/10.1111/j.1095-8339.1989.tb00167.x>

Oliver EGH (1990) New species of *Erica* (Ericaceae) from the Cape Province. Bothalia 20: 41–48. <https://doi.org/10.4102/abc.v20i1.892>

Oliver EGH (1991) The Ericoideae (Ericaceae) – a review. Contributions from the Bolus Herbarium 13: 158–208.

Oliver EGH, Oliver IM (1991) Studies in the Ericoideae (Ericaceae). VIII. New species in *Erica*, section *Pseuderemia* , from southern Africa. Bothalia 21: 137–142. <https://doi.org/10.4102/abc.v21i2.873>

Oliver EGH (1992) Studies in the Ericoideae (Ericaceae). VIII [recte IX]. New combinations for *Philippia* are made in *Erica* for the Flora Zambesiaca region. Kew Bulletin 47: 665–668. <https://doi.org/10.2307/4110704>

Schumann D, Kirsten G, Oliver EGH (1992) Ericas of South Africa. Fernwood Press, Vlaeberg, South Africa, 272 pp.

Linder HP, Vlok JH, McDonald DJ, Oliver EGH, Boucher C, Van Wyk BE, Schutte A (1993) The high altitude flora and vegetation of the Cape Floristic Region, South Africa. Opera Botanica 121: 247–261. Available from: <http://www.ben-erikvanwyk.com/66%20-%201993,%20Linder,%20Vlok,%20McDonald,%20Oliver,%20Boucher,%20Van%20wyk,%20Schutte.pdf> (November 10, 2023).

Oliver EGH (1993a) Studies in the Ericoideae (Ericaceae). X. Nomenclatural Changes for the Flore des Mascareignes Region. Kew Bulletin 48: 767–769. <https://doi.org/10.2307/4118855>

Oliver EGH (1993b) Studies in the Ericoideae (Ericaceae). XI. The generic relationship between *Erica* and *Blaeria*. Kew Bulletin 48: 771–780. <https://doi.org/10.2307/4118856>

Oliver EGH (1993c) Studies in the Ericoideae (Ericaceae). XII. The placing of the genus *Blaeria* into synonymy under *Erica*; nomenclatural and taxonomic changes for the southern African region. Bothalia 23: 1–7. <https://doi.org/10.4102/abc.v23i1.779>

Oliver EGH (1993d) Studies in the Ericoideae (Ericaceae). XIII. Three new species of *Erica* from the southwestern Cape. Bothalia 23: 9–14. <https://doi.org/10.4102/abc.v23i1.780>

Oliver EGH (1994a) Phytogeography and endemism in the Ericoideae (Ericaceae). Proc. XIIIth Plenary Meeting AETFAT, Malawi 2: 941–951.

Oliver EGH (1994b) Studies in the Ericoideae (Ericaceae). XV. The generic relationship between *Erica* and *Ericinella*. Bothalia 24: 121–126. <https://doi.org/10.4102/abc.v24i2.761>

Oliver EGH, Oliver IM (1994) Studies in the Ericoideae (Ericaceae). XIV. Notes on the genus *Erica*. Bothalia 24: 25–30. <https://doi.org/10.4102/abc.v24i1.746>

Oliver EGH (1995) *Erica* – an update on species numbers. Yearbook of the Heather Society: 11–12. Available from: <https://www.biodiversitylibrary.org/page/43695553>.

Oliver EGH (1995) Ericaceae. A new species of *Erica* from the Western Cape. Bothalia 25: 242–244. <https://doi.org/10.4102/abc.v25i2.735>

Oliver EGH, Oliver IM (1995) Studies in the Ericoideae (Ericaceae). XVI. Six new species of *Erica* from the Western Cape, South Africa. Bothalia 25: 87–95. <https://doi.org/10.4102/abc.v25i1.714>

Oliver EGH (1996) The position of *Bruckenthalia* versus *Erica*. Yearbook of the Heather Society: 6. Available from: <https://www.biodiversitylibrary.org/page/43695628>.

Oliver EGH, Oliver IM (1996a) Studies in the Ericaceae (Ericoideae) XIX. Two new species of *Erica* from southern Africa. Feddes Repertorium 107: 305–310. <https://doi.org/10.1002/fedr.19961070506>

Oliver EGH, Oliver IM (1996b) Studies in the Ericaceae (Ericoideae) XVIII. Two new species of *Erica* from the southern part of South Africa. Feddes Repertorium 106: 347–352. <https://doi.org/10.1002/fedr.19961060505>

Oliver EGH, Oliver IM (1996c) Studies in the Ericaceae (Ericoideae), XX. A rare new species of *Erica* from South Africa. Yearbook of the Heather Society: 1–5. Available from: <https://www.biodiversitylibrary.org/itemdetails/148471>.

Oliver EGH, Oliver IM (1997) A new species of *Erica* from the Western Cape. Bothalia 27: 142–144. <https://doi.org/10.4102/abc.v27i2.675>

Oliver EGH, Oliver IM, Volk FW (1997) Studies in the Ericaceae (Ericoideae), XXI. *Erica oakesiorum*, a new tree species from South Africa. Yearbook of the Heather Society: 13–18. Available from: <https://www.biodiversitylibrary.org/page/43695795>.

Oliver EGH (1998) Erica, a biodiversity record for plants? Yearbook of the Heather Society: 1–2. Available from: <https://www.biodiversitylibrary.org/page/43695863>.

Oliver EGH, Oliver IM (1998a) A new species of *Erica* (Ericaceae) from South Africa. Novon 8: 430–432. <https://doi.org/10.2307/3391868>

Oliver EGH, Oliver IM (1998b) *Erica schumannii*, a new mat-forming species from South Africa. Yearbook of the Heather Society: 32–38. Available from: <https://www.biodiversitylibrary.org/page/43695894>.

Oliver EGH, Oliver IM (1998c) Three new species of *Erica* (Ericaceae) from South Africa. Novon 8: 267–274. <https://doi.org/10.2307/3392018>

Dorr LJ, Oliver EGH (1999a) A new combination in *Erica* (Ericaceae) from São Tomé. Kew Bulletin 54: 235–236. Available from: <http://www.jstor.org/stable/4111048>.

Dorr LJ, Oliver EGH (1999b) New taxa, names, and combinations in *Erica* (Ericaceae-Ericoideae) from Madagascar and the Comoro Islands. Adansonia 21: 75–91. Available from: <https://sciencepress.mnhn.fr/fr/periodiques/adansonia/21/1/nouveaux-taxons-noms-et-combinaisons-dans-le-genre-erica-ericaceae-ericoideae-madagascar-et-aux-comores> (May 12, 2023).

Oliver EGH (1999) Systematic Studies in the Tribe Ericeae (Ericaceae-Ericoideae). Doctoral dissertation. University of Cape Town

Oliver EGH, Oliver IM (1999a) *Erica hanekomii*, a new prostrate species from the Western Cape, South Africa. Yearbook of the Heather Society: 36–42. Available from: <https://www.biodiversitylibrary.org/page/43695741>.

Oliver EGH, Oliver IM (1999b) Ericaceae. Three new species of *Erica* from Western Cape, South Africa. Bothalia 29: 113–117. <https://doi.org/10.4102/abc.v29i1.583>

Oliver EGH, Oliver IM (1999c) Ericaceae. Two new species of *Erica* from Western Cape South Africa. Bothalia 29: 95–98. <https://doi.org/10.4102/abc.v29i1.575>

Oliver EGH (2000a) An obsesssion with *Erica*. Yearbook of the Heather Society: 52–56. Available from: <https://www.biodiversitylibrary.org/page/43695473>.

Oliver EGH (2000b) Systematics of Ericaceae (Ericeae-Ericoideae): species with indehiscent and partially dehiscent fruits. Contributions from the Bolus Herbarium 19.

Oliver EGH, Oliver IM (2000a) *Erica ignita*, a new showy species from South Africa. Yearbook of the Heather Society: 63–68. Available from: <https://www.biodiversitylibrary.org/page/43695484>.

Oliver EGH, Oliver IM (2000b) *Erica kirstenii*, a new rock-loving species from South Africa. Yearbook of the Heather Society: 57–62. Available from: <https://www.biodiversitylibrary.org/page/43695478>.

Oliver I, Oliver T [EGH] (2000c) Field guide to the ericas of the Cape Peninsula. Protea Atlas Project, National Botanical Institute.

Oliver EGH, Oliver IM (2000d) Ericaceae. Two new species of *Erica* from Western Cape, South Africa. Bothalia 30: 49–53. <https://doi.org/10.4102/abc.v30i1.539>

Oliver EGH, Oliver IM (2000e) Three new species of *Erica* (Ericaceae) from Western Cape, South Africa. Bothalia 30: 147–153. <https://doi.org/10.4102/abc.v30i2.552>

Oliver EGH, Oliver IM (2000f) Ericaceae. In: Goldblatt P, Manning J (Eds), Cape plants: a conspectus of the Cape flora of South Africa, Strelitzia 9. National Botanical Institute, 423–452. Available from: <https://www.cabdirect.org/cabdirect/abstract/20013066367> (October 4, 2023).

Oliver EGH, Oliver IM (2001a) Taxonomic problems in the *Erica filipendula* complex. Yearbook of the Heather Society: 27–34. Available from: <https://www.biodiversitylibrary.org/page/43784659>.

Oliver EGH, Oliver IM (2001b) The ultimate prize – a new species of *Erica*! Yearbook of the Heather Society: 9–14. Available from: <https://www.biodiversitylibrary.org/page/43784641>.

Oliver EGH, Oliver IM (2001c) Five new species of *Erica* (Ericaceae) from the Swartberg Range, Western Cape, South Africa and a note on E. esterhuyseniae. Bothalia 31: 155–165. <https://doi.org/10.4102/abc.v31i2.514>

Oliver EGH, Oliver IM (2001d) Four new species of *Erica* (Ericaceae) from Western Cape, South Africa. Bothalia 31: 1–8. <https://doi.org/10.4102/abc.v31i1.493>

Cafferty S, Oliver EGH, Oliver I (2002) (1561-1563) Proposals to conserve the names *Erica calycina*, *E. corifolia*, and *E. imbricata* (Ericaceae) with conserved types. Taxon 51: 810–812. <https://doi.org/10.2307/1555049>

Oliver EGH, Oliver IM (2002a) A new rock-loving species of *Erica* from the eastern Swartberg, South Africa. Yearbook of the Heather Society: 31–36. Available from: <https://www.biodiversitylibrary.org/page/43784981>.

Oliver EGH, Oliver IM, Volk FW, Forshaw N (2002) Genus *Erica* Interactive Identification Key V.1.00. <https://doi.org/10.5281/zenodo.10354934>

Cleevely RJ, Oliver EGH (2002) A preliminary note on the publication dates of H. C. Andrews’ Coloured engravings of heaths (1794–1830). Archives of Natural History 29: 245–264. <https://doi.org/10.3366/anh.2002.29.2.245>

Oliver EGH, Oliver IM (2002b) The genus *Erica* (Ericaceae) in southern Africa: taxonomic notes 1. Bothalia 32: 37–61. <https://doi.org/10.4102/abc.v32i1.461>

Oliver EGH, Oliver IM (2002c) Six new species and one new subspecies of *Erica* (Ericaceae) from Western Cape, South Africa. Bothalia 32: 167–180. <https://doi.org/10.4102/abc.v32i2.480>

Oliver EGH (2003) Footnote [to A. Hitchcock, *Erica verticillata* is brought back from the brink of extinction. Yearbook of The Heather Society 2003: 45–50]. Yearbook of the Heather Society: 50. Available from: <https://www.biodiversitylibrary.org/page/43784760>.

Cleevely RJ, Nelson EC, Oliver EGH (2003) More accurate publication dates for H. C. Andrews’ The Heathery, particularly volumes 5 and 6. Bothalia 33: 195–198. <https://doi.org/10.4102/abc.v33i2.452>

Nelson EC, Oliver EGH (2003) Understanding *Erica* x *willmorei*, a nineteenth century English garden hybrid. Bothalia 33: 149–154. <https://doi.org/10.4102/abc.v33i2.443>

Oliver EGH (2004a) *Erica*. In: Kubitzki K (Ed.), The families and genera of vascular plants, volume 6. Flowering Plants. Dicotyledons: Celastrales, Oxalidales, Rosales, Cornales, Ericales. Springer Berlin Heidelberg, Berlin, Heidelberg. <https://doi.org/10.1007/978-3-662-07257-8_19>

Oliver EGH (2004b) Ericaceae. Nomenclatural changes in *Erica*. Bothalia 34: 38. <https://doi.org/10.4102/abc.v34i1.408>

Oliver EGH, Oliver IM (2004) Two new species of *Erica* (Ericaceae); one from Western Cape and one from KwaZulu-Natal, South Africa. Bothalia 34: 11–15. <https://doi.org/10.4102/abc.v34i1.400>

Turner RC, Oliver EGH (2004) Ericaceae. A new species of indehiscent-fruited *Erica* from the central Kouebokkeveld, Western Cape, South Africa. Bothalia 34: 39–41. <https://doi.org/10.4102/abc.v34i1.409>

Nelson EC, Oliver EGH (2004) Cape heaths in European gardens: the early history of South African *Erica* species in cultivation, their deliberate hybridization and the orthographic bedlam. Bothalia 34: 127–140. <https://doi.org/10.4102/abc.v34i2.427>

Nelson EC, Oliver EGH (2005) Chromosome numbers in *Erica* - an updated checklist. Heathers 2: 57–58. Available from: <https://www.biodiversitylibrary.org/page/43784163> (November 10, 2023).

Oliver EGH (2005) [Photograph & caption] *Erica junonia* var. *junonia*. Heathers: ii. Available from: <https://www.cabdirect.org/cabdirect/abstract/20053124100> (November 10, 2023).

Volk FW, Forshaw N, Schumann D, Oliver EGH, Oliver IM (2005) Genus *Erica* Interactive Identification Key V.2.00. Contributions from the Bolus Herbarium 22. <https://doi.org/10.5281/zenodo.10362198>

Oliver EGH, Oliver IM (2005) The genus *Erica* (Ericaceae) in southern Africa: taxonomic notes 2. Bothalia 35: 121–148. <https://doi.org/10.4102/abc.v35i2.388>

Oliver EGH (2006) *Erica amidae*, a new rock-dwelling species near Cape Town, South Africa. Heathers 3: 22–29. Available from: <https://www.biodiversitylibrary.org/page/43785052>.

Turner RC, Oliver EGH (2006) Two new species of *Erica* (Ericaceae) from the Langeberg, Western Cape, South Africa. Bothalia 36: 33–37. <https://doi.org/10.4102/abc.v36i1.329>

Oliver EGH (2007) Linnaeus’ Cape species of *Erica*. Heathers 4: 5–10. Available from: <https://www.biodiversitylibrary.org/page/43784094>.

Oliver EGH (2008) Old painting instrumental in rediscovery of a Cape *Erica* species. Heathers 5: 39–43. Available from: <https://www.biodiversitylibrary.org/page/43784909>.

Oliver EGH (2009) [Photograph & caption] *Erica patens*. Heathers 6: ii. Available from: <https://www.biodiversitylibrary.org/page/43784790>.

Oliver EGH (2010) The long-lost *Erica greyi*. Heathers 7: 13–15. Available from: <https://www.biodiversitylibrary.org/page/43785123>.

Oliver EGH (2011) Hunting for Ericas in Madagascar. Heathers 8: 47–54. <https://doi.org/10.5281/zenodo.10361920>

Pirie MD, Oliver EGH, Bellstedt DU (2011) A densely sampled ITS phylogeny of the Cape flagship genus *Erica* L. suggests numerous shifts in floral macro-morphology. Molecular Phylogenetics and Evolution 61: 593–601. <https://doi.org/10.1016/j.ympev.2011.06.007>

Oliver EGH (2012) Ericaceae. In: Manning JC, Goldblatt P (Eds), Plants of the Greater Cape Floristic Region, Volume 1: The Core Cape Flora. Strelitzia. South African National Biodiversity Institute (SANBI Publishing), Pretoria, 482—511. Available from: <https://www.sanbi.org/wp-content/uploads/2018/04/strelitzia-29-2012.pdf>.

Oliver EGH, Forshaw N (2012) Genus *Erica* An Identification Aid Version 3.00. Contributions from the Bolus Herbarium 23. <https://doi.org/10.5281/zenodo.10362291>

Hitchcock AN, Oliver EGH, Thomas V (2013) *Erica verticillata*: Ericaceae. Flowering Plants of Africa 63: 104–119. Available from: <https://www.cabdirect.org/cabdirect/abstract/20163287173> (November 10, 2023).

Oliver EGH (2014) Ericas in Mauritius, home of the dodo. Heathers 11: 38–42. <https://doi.org/10.5281/zenodo.10361982>

Mugrabi de Kuppler AL, Fagúndez J, Bellstedt DU, Oliver EGH, Léon J, Pirie MD (2015) Testing reticulate versus coalescent origins of *Erica lusitanica* using a species phylogeny of the northern heathers (Ericeae, Ericaceae). Molecular Phylogenetics and Evolution 88: 121–131. <https://doi.org/10.1016/j.ympev.2015.04.005>

Oliver EGH (2015) Looking for Spanish heathers. Heathers 12: 27–34. <https://doi.org/10.5281/zenodo.1036202>

Pirie MD, Oliver EGH, Mugrabi de Kuppler A, Gehrke B, Le Maitre NC, Kandziora M, Bellstedt DU (2016) The biodiversity hotspot as evolutionary hot-bed: spectacular radiation of *Erica* in the Cape Floristic Region. BMC Evolutionary Biology 16: 190. <https://doi.org/10.1186/s12862-016-0764-3>

Pirie MD, Oliver EGH, Gehrke B, Heringer L, Mugrabi de Kuppler A, Le Maitre NC, Bellstedt DU (2017) Underestimated regional species diversity in the Cape Floristic Region revealed by phylogenetic analysis of the *Erica abietina*/*E. viscaria* clade (Ericaceae). Botanical Journal of the Linnean Society 184: 185–203. <https://doi.org/10.1093/botlinnean/box021>

Pirie MD, Kandziora M, Nürk NM, Le Maitre NC, Mugrabi de Kuppler A, Gehrke B, Oliver EGH, Bellstedt DU (2019) Leaps and bounds: geographical and ecological distance constrained the colonisation of the Afrotemperate by *Erica*. BMC Evolutionary Biology 19: 222. <https://doi.org/10.1186/s12862-019-1545-6>

Nelson EC, Oliver EGH, Pirie MD (2023) *Erica* L. (Ericaceae): homonyms amongst published names for African species and proposed replacement names. PhytoKeys 236: 157–178. <https://doi.org/10.3897/phytokeys.236.110498>

Oliver EGH, Forshaw N, Oliver IM, Volk F, Schumann AWS, Dorr LJ, Hoekstra RD, Musker SD, Nürk NM, Pirie MD, Rebelo AG (2024) Genus Erica: An identification aid version 4.00. PhytoKeys 241: 143–154. <https://doi.org/10.3897/phytokeys.241.117604>

Pirie M, Bellstedt D, Bouman R, Fagúndez J, Gehrke B, Kandziora M, Maitre NCL, Musker S, Newman E, Nürk N, Oliver EGH, Pipins S, Niet T van der, Forest F (2024) Spatial decoupling of taxon richness, phylogenetic diversity, and threat status in the megagenus Erica. ARPHA Preprints 5: e124629. <https://doi.org/10.3897/arphapreprints.e124629>
